# *Nocardia otitidiscaviarum* meningitis in an immunocompetent patient diagnosed by metagenomic next-generation sequencing: a case report

**DOI:** 10.3389/fmed.2025.1588977

**Published:** 2025-08-11

**Authors:** Ping Chen, Juan Li, Ping-Xian Liu, Hui Gou, Ming Yu, Lin-Lin Li, Luwen Huang

**Affiliations:** ^1^Department of Pharmacy, Suining Central Hospital, Suining, China; ^2^Laboratory of Human Diseases and Immunotherapy, West China Hospital, Sichuan University, Chengdu, China; ^3^Department of Pharmacy, Southwest Medical University, Luzhou, China; ^4^Department of Neurology, Suining Central Hospital, Suining, China

**Keywords:** *Nocardia otitidiscaviarum*, purulent meningitis, infection, metagenomic next-generation sequencing, antimicrobial therapy

## Abstract

Cerebral nocardiosis caused by *Nocardia* represents a rare and diagnostically challenging infectious disease, predominantly affecting immunocompromised patients. This opportunistic infection may also pose life-threatening risks to immunocompetent individuals. The diagnostic process is frequently complicated by the absence of distinctive clinical manifestations and technical limitations inherent to conventional microbiological detection methods, which collectively impede the acquisition of definitive pathogenic evidence, thereby resulting in diagnostic delays. This case report describes a 67-year-old immunocompetent male bricklayer who presented with recurrent febrile episodes during hospitalization and was ultimately diagnosed with purulent meningitis based on clinical history corroborated by cerebrospinal fluid (CSF) analytical findings. Ceftriaxone was initially employed as an anti-infective agent, however, it was ineffective. Consequently, the treatment was escalated to a combination of meropenem and vancomycin, yet the patient’s condition did not significantly improve. Concurrently, repeated cultures of the patient’s blood and CSF yielded no identifiable pathogens. Notably, three months ago, the patient accidentally sustained a laceration on the left thigh by an unknown object during work. An abscess gradually developed at the site of the laceration, and incision and drainage were carried out at a local hospital. However, the wound did not heal satisfactorily after the surgery, raising concerns about potential rare pathogenic bacterial infections. Ultimately, the pathogen was successfully identified as *Nocardia otitidiscaviarum* through metagenomic next-generation sequencing (mNGS). Following this diagnosis, the patient’s condition was rapidly controlled after initiating treatment with the targeted drug combination of sulfamethoxazole, meropenem, and amikacin. Given the high misdiagnosis rate and poor sensitivity of cultures for *Nocardia otitidiscaviarum* in cases of intracranial infections, this case underscores the critical role of mNGS in the diagnosis and selection of effective antibiotics for treating *Nocardia* intracranial infections.

## Introduction

*Nocardia* is a type of aerobic, gram-positive bacterium that is typically located in soil, dust, decomposing plant material, and is generally regarded as an uncommon opportunistic pathogen (1). While immunocompromised individuals are widely believed to be particularly susceptible to *Nocardia* infections, it is noteworthy that approximately one-third of cases occur in immunocompetent individuals (2, 3). *Nocardia* can lead to either localized or systemic suppurative diseases (4). Although central nervous system (CNS) involvement is a rare manifestation of invasive *Nocardia* disease, the associated mortality rate can be as high as 50% (5). Purulent meningitis caused by *Nocardia* is rarely reported, which may lead to misdiagnosis. The difficulties in identifying *Nocardia* and the resulting delays in commencing effective anti-infective therapy can have catastrophic consequences (2, 6). In this report, we describe a case of purulent meningitis attributed to *Nocardia otitidiscaviarum*, which was accurately diagnosed using mNGS. Through swift and precise diagnostics, the patient was given personalized treatment, leading to significant improvement in his condition.

## Case report

**Figure 1:**
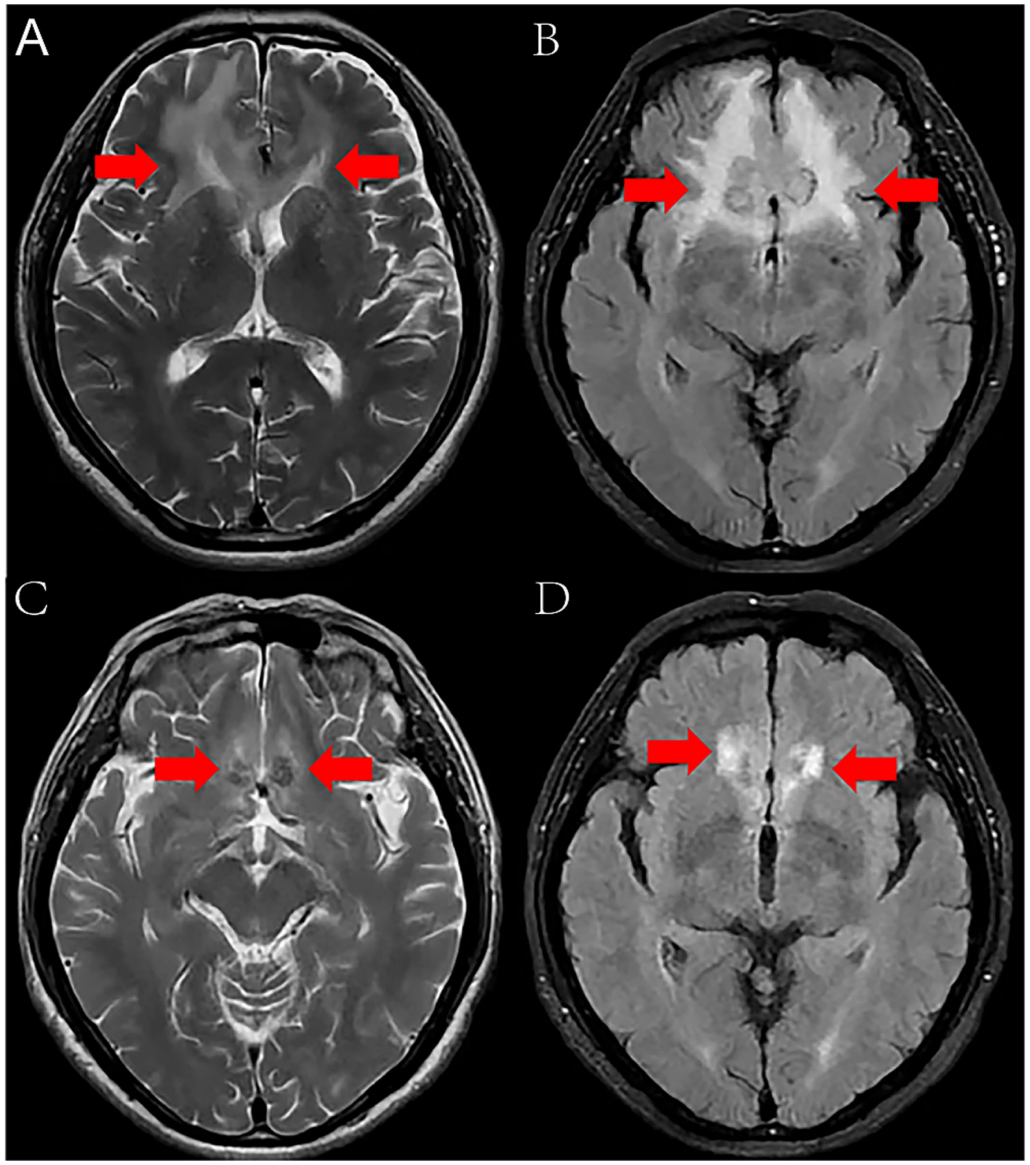
MRI revealed bilateral frontal lobe lesions exhibiting irregular patchy morphology, displaying hyperintense signals on T2WI and FLAIR **(A,B)**, MRI demonstrated slightly hyperintense signals on T2WI and FLAIR **(C,D)**, lesional volume showed significant reduction after 11-month treatment.

On January 27, 2024, a 67-year-old male bricklayer presented to the Department of Neurology at Suining Central Hospital with a 10-day history of progressive headache accompanied by nausea and vomiting. The patient had no significant medical history of hypertension, diabetes mellitus, or immunodeficiency disorders. He denied any known allergies to medications or food products. There was no history of recent travel or exposure to animals. Three months ago, the patient accidentally sustained a laceration on the left thigh by an unknown object during work. Due to tolerable wound pain, he did not pay sufficient attention and continued working at the construction site for several days. Unfortunately, the wound site gradually developed into a localized abscess. He underwent incision and drainage surgery for a left thigh abscess at another hospital, however, the wound failed to heal after the procedure. On admission, physical examination revealed: body temperature of 36.7°C, pulse at 82 beats per minute, respiration at 20 breaths per minute, blood pressure measuring 117/81 mmHg, and heart rate at 82 beats per minute. Clear breath sounds in both lungs without audible dry or moist rales. The heart rhythm was regular, and no pathological murmurs were detected in any valvular auscultation areas. The neck was supple without rigidity, and both Kernig’s sign and Brudzinski’s sign were negative. Laboratory tests indicated a white blood cell count of 9.4 × 10^9/L (normal range 3.5–9.5 × 10^9/L), a neutrophil granulocyte count of 5.28 × 10^9/L (normal range 1.8–6.3 × 10^9/L), a C-reactive protein level of 7.86 mg/L (normal range <10 mg/L), and an albumin concentration of 47.2 g/L. The Magnetic resonance imaging (MRI) revealed bilateral frontal lobe lesions exhibiting irregular patchy morphology, displaying hyperintense signals on T2-weighted imaging (T2WI) and Fluid-Attenuated Inversion Recovery (FLAIR) sequences (Figures 1A,B), suggestive of potential infectious or occupying lesions. Considering the patient’s history of headaches, along with the results from physical examinations and laboratory markers indicative of inflammation, we preliminarily consider the patient to have headache (etiology undetermined). Treatment included the administration of loxoprofen sodium tablets (60 mg tid) for pain relief, and intravenous infusions of mannitol (25 g bid) and glycerol fructose (250 mL bid) were alternated on day 1 to promote dehydration and reduce intracranial pressure. Two days post-treatment, the patient developed a high-grade fever, reaching a peak body temperature of 39.2°C, accompanied by chills, aggravated headache, nausea, vomiting, and neck stiffness. On the same day, a lumbar puncture was conducted, revealing a pressure of 420 mmH_2_O (normal range 80–180 mmH_2_O). CSF appeared light yellow and slightly turbid. CSF analysis revealed white blood cellcount (WBC) of 3,618 cells/μL (normal range 0–5 cells/μL), a glucose level of 3.20 mmol/L (normal range 2.5–4.5 mmol/L), and a protein level of 3.21 g/L (normal range 0.15–0.45 g/L). CSF samples underwent comprehensive laboratory testing, including Gram stain, India ink stain, acid-fast stain, *Mycobacterium tuberculosis* TB-DNA detection, herpes simplex virus type 1 DNA quantification, and herpes simplex virus type 2 DNA quantification, all yielding negative results. Following comprehensive evaluation by the multidisciplinary team (MDT), the consensus opinion was that the patient had headache, vomiting and high fever, but no symptoms of tuberculosis poisoning (low fever, night sweats, loss of appetite, etc.) or cranial nerve involvement symptoms (ptosis, diplopia, facial paralysis), the acid-fast stain and TB-DNA of *Mycobacterium tuberculosis* were negative, therefore, tuberculous meningitis was temporarily not considered. Although the patient exhibited rapid disease progression with fever, headache, nausea, vomiting, and meningeal irritation signs, the CSF analysis revealed WBC of 3,618 cells/μL and elevated protein level (3.21 g/L), herpes simplex virus type 2 DNA quantification was negative, viral meningitis was deemed unlikely in this clinical context. The patient was immunocompetent with no history of pigeon exposure or prolonged residence in pigeon-excrement-contaminated environments and his CSF India ink was negative, fungal meningitis was considered unlikely. Ultimately, the MDT integrated the patient’s medical history, clinical manifestations, and laboratory findings to conclude that purulent meningitis was the most probable diagnosis. At the same time, the MDT recommended conducting the G-(1,3)-β-D-glucan test (G test), the galactomannan test (GM test), blood culture, and CSF culture, suggesting that rare pathogen infections should be considered in cases of inadequate response to antimicrobial therapy. Antibacterial treatment with ceftriaxone (2 g q12h) was empirically initiated on day 3. Despite this intervention, the patient continued to experience recurrent high fever with no improvement in symptoms. Consequently, on day 5, meropenem (2.0 g q8h) and vancomycin (0.5 g q6h) were empirically added to enhance anti-infection measures. Subsequent tests, including the G test, GM test, blood culture, and CSF culture, all returned negative results.

**Figure 2:**
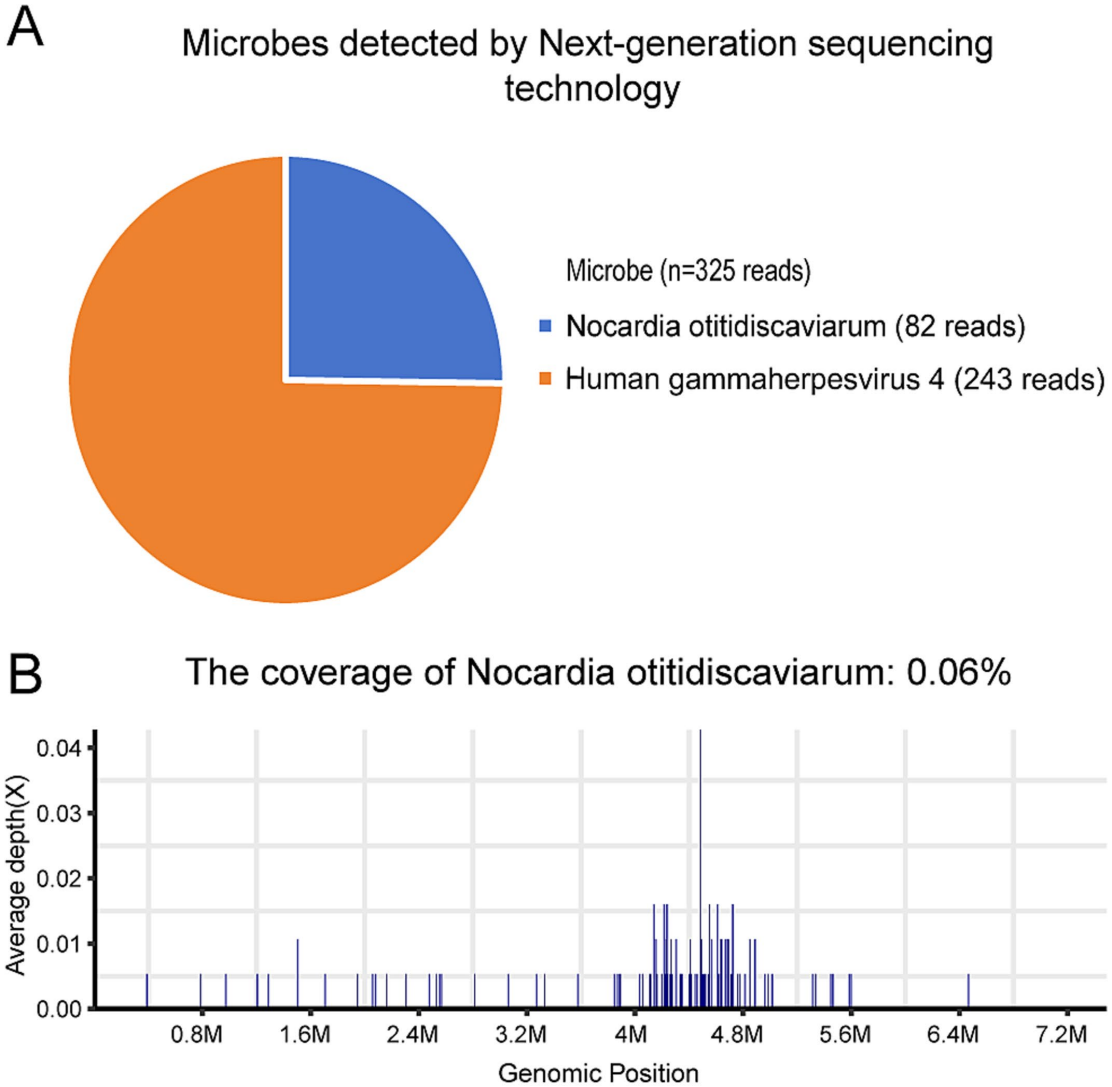
*Nocardia otitidiscaviarum* in cerebrospinal fluid was detected by mNGS. **(A)** Microbes detected by mNGS; **(B)** The coverage of *Nocardia otitidiscaviarum*.

CSF sample was performed to identify the pathogens on day 5. DNA was extracted from all samples using a QIAamp® UCP Pathogen DNA Kit (Qiagen) according to the manufacturer**’**s instructions. Human DNA was removed using Benzonase (Qiagen) and Tween20 (Sigma). The QIAamp UCP pathogen mini kit (Qiagen, Valencia, CA, USA) was applied to extract total RNA. RNA was reversely transcribed and amplified by the Ovation RNA-Seq system (NuGEN, CA, USA). Libraries were constructed for DNA using the Nextera XT DNA Library Prep Kit (Illumina, San Diego, CA). The library quality was assessed using the Qubit dsDNA HS Assay kit followed by a high-sensitivity DNA kit (Agilent) on the Agilent 2,100 Bioanalyzer. Library pools were then loaded onto the Illumina NextSeq 550Dx sequencer for 75 cycles of single-end sequencing to generate approximately 20 million reads for each library. High-quality sequencing data were generated by removing low-quality reads, followed by computational subtraction of human host sequences mapped to the human reference genome (hg19) using Burrows-Wheeler alignment. The remaining data by removal of low-complexity reads were classified by simultaneously aligning to four microbial genome databases (bacteria, fungi, viruses and parasites), which were downloaded from the National Center for Biotechnology Information (ftp://ftp.ncbi.nlm.nih.gov/genomes/). Bacterial and virus were identified as suspected pathogens when number of sequences strictly mapped at species level (SMRN) of microorganisms in sample was higher than in the negative control and≥3 simultaneously. *Nocardia otitidiscaviarum* (82 reads), and *human gammaherpesvirus* 4 (243 reads) were detected (Figures 2A,B).

**Figure 3:**
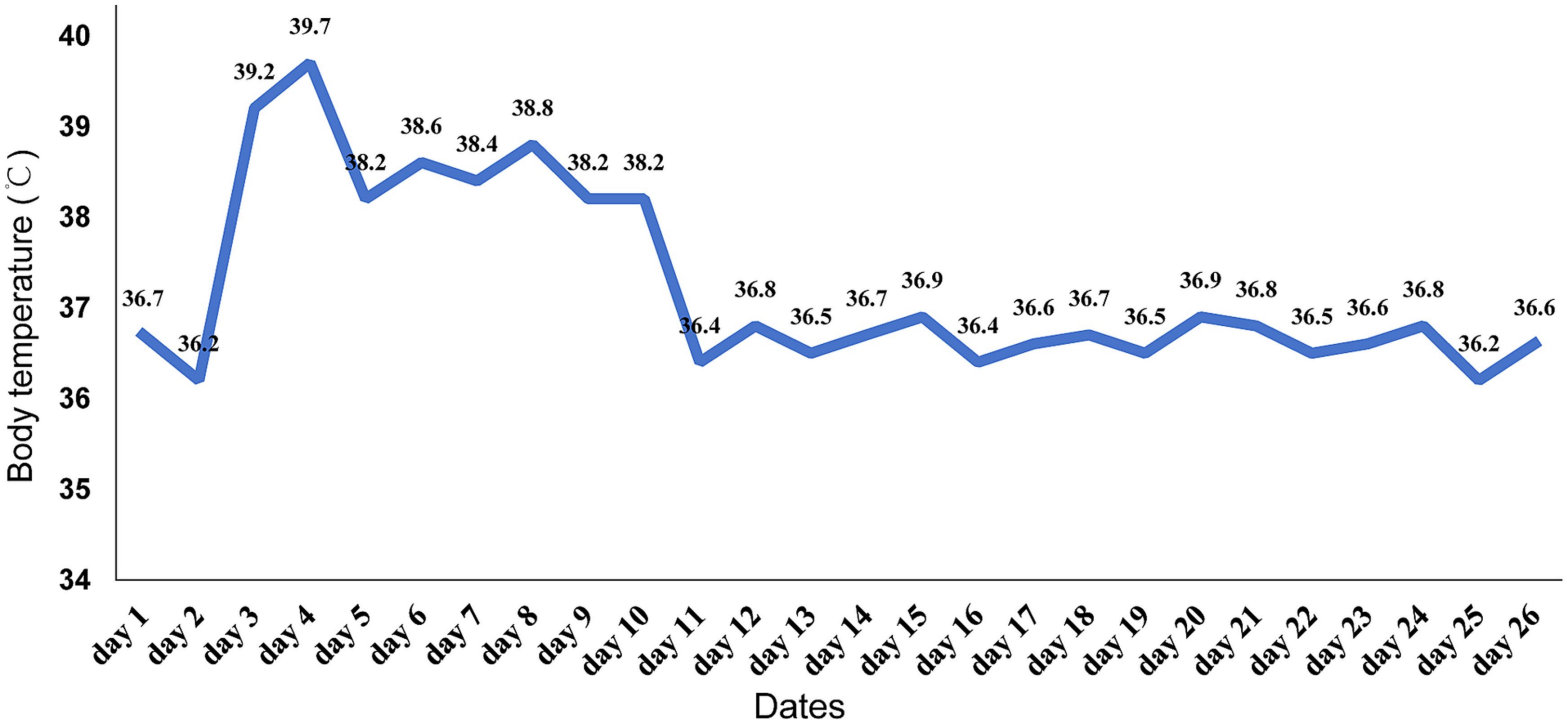
Patient’s temperature changes during hospitalization.

**Figure 4:**
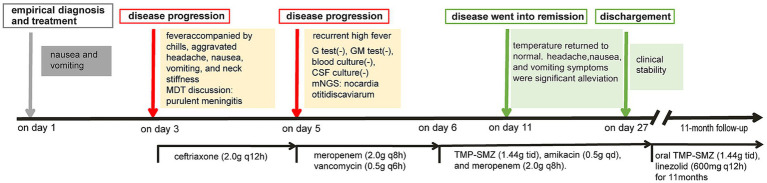
The patient’s timeline of antimicrobial therapy.

Based on the patient’s demographic characteristics (a 67-year-old male bricklayer), medical history, CSF biochemical routine results, mNGS findings, and clinical manifestations, a definitive diagnosis of *Nocardia otitidiscaviarum*-induced purulent meningitis was ultimately established. On day 6, the antimicrobial regimen was optimized to include trimethoprim-sulfamethoxazole (TMP-SMZ) (1.44 g tid), amikacin (0.5 g qd), and meropenem (2.0 g q8h). Following this therapeutic adjustment, the patient demonstrated significant clinical improvement. By day 11, the patient’s temperature returned to normal (Figure 3), headache gradually subsided, with a marked reduction in episodes of nausea and vomiting. Concurrently, CSF analysis revealed substantial improvement in both biochemical and cytological parameters. On day 27, the patient exhibited clinical stability and requested discharge. It was recommended that the patient be transferred to a local hospital to continue receiving combination antimicrobial therapy with meropenem, TMP-SMZ, and amikacin for an additional 14 days, followed by switching to oral TMP-SMZ and linezolid. The total duration of antimicrobial therapy should be at least 12 months to ensure adequate treatment completion. During the 11-month post-discharge follow-up evaluation, the patient maintained stable clinical status. After completing a 12-month course of antimicrobial therapy, antimicrobial agents were discontinued with no evidence of relapse. The patient expressed satisfaction with the therapeutic outcome and had resumed full occupational and daily activities. Follow-up CSF analysis demonstrated complete normalization (Table 1). Follow-up MRI demonstrated slightly hyperintense signals on T2WI and FLAIR, lesional volume showed significant reduction after 11-month treatment, indicating a favorable response to antimicrobial therapy (Figures 1C,D). The timeline of antimicrobial therapy is illustrated in Figure 4.

**Table 1:** Patient’s CSF test results.

Category	Laboratory tests	Normal range	Results					
			On day 3	On day 9	On day 24	Discharge for 1 month	Discharge for 2 months	Discharge for 11 months
Appearance and pressure	Appearance	Clear and transparent	Light yellow slightly turbid	Light yellow slightly turbid	Light yellow slightly turbid	Clear and transparent	Clear and transparent	Clear and transparent
	Pressure (mmH_2_O)	80 ~ 180	420	350	210	152	153	147
Inflammatory markers	WBC (cells/μL)	0 ~ 5	3,618	1,363	680	75	60	6
	Protein quantification (g/L)	0.15 ~ 0.45	3.21	5.01	0.86	0.76	0.73	0.57
Metabolic values	Chloride (mmol/L)	120 ~ 132	121	114	117	126	127	127
	Glucose (mmol/L)	2.4 ~ 4.5	3.21	1.7	2.5	2.6	2.0	3.4

## Discussion

*Nocardia*, an opportunistic pathogen, was first isolated in 1888 (7). The incidence of *Nocardia* disease ranging from 0.33 to 0.87 per 100,000 individuals. This incidence is particularly high among middle-aged and elderly populations, with males being affected at a rate three times greater than that of females (8). *Nocardia* can be inhaled by hosts and is well known for causing life-threatening infections in humans, particularly those affecting the lungs, skin, soft tissues, the intracranial and abdominal cavities. Notably, *Nocardia* affects the lungs more frequently than the CNS. The genus *Nocardia* comprises over 80 species, of which more than 50 are capable of causing disease in humans (9). Among the isolated strains, the most prevalent isolates responsible for human disease were *Nocardia asteroides*, *Nocardia brasiliensis*, *Nocardia otitidiscaviarum*, and *Nocardia mallei*. Different species of *Nocardia* exhibit varying sensitivities to antibiotics, underscoring the critical importance of accurate bacterial species identification. Investigators conducting a retrospective analysis of *Nocardia* infections in Southwestern China over the past decade demonstrated that *Nocardia* exhibited 100% susceptibility rates to amikacin, linezolid, and tetracyclines. This was followed by TMP-SMZ (90.9%), gentamicin (75.0%), and carbapenems (70.0%), while lower susceptibility rates were observed for fluoroquinolones and penicillin (10). Suining Central Hospital, where we are based, is a tertiary care institution located in Southwest China.

*Nocardia* primarily invades the human body through inhalation or skin wounds (11, 12). Following this initial invasion, *Nocardia* can disseminate via the bloodstream to the CNS and other regions of the body (13). *Nocardia* infections can occasionally occur in immunocompetent individuals (5, 14), those who are immunosuppresse—such as individuals with human immunodeficiency virus (HIV) infection, those receiving long-term hormone therapy, recipients of stem cell or solid organ transplants, and patients with malignant tumors—are at increased risk of infection (15, 16). Additionally, chronic lung disease, diabetes, and tuberculosis are among the most common underlying conditions associated with CNS *Nocardia* infections (17, 18). When *Nocardia* invades and leads to intracranial infection, it is referred to as cerebral *nocardiosis*. This condition often presents with seizures, motor weakness, headache, fever, nausea, and vomiting, however, these clinical symptoms are nonspecific (19, 20). The imaging characteristics of cerebral *nocardiosis* are also nonspecific, often appearing as single or multiple lesions. A study of 89 cases of cerebral *Nocardiosis* revealed that 93% of the patients’ head images revealed circular enhanced lesions, with 50% exhibiting multiple lesions (8). Such imaging features can easily be misdiagnosed as typical intracranial infections, particularly when empirical anti-infective therapy has proven ineffective. Consequently, the diagnosis of cerebral *nocardiosis* is challenging. The patient had no underlying comorbidities, had not received glucocorticoid therapy, and lacked evidence of immunodeficiency or chronic pulmonary disease. As a bricklayer, he sustained a skin cut at a construction site three months prior, which resulted in pustule formation in the affected area. Therefore,we hypothesize that the *Nocardia* infection was acquired through cutaneous lesions and hematogenously disseminated to the brain.

Traditional techniques for diagnosing *Nocardia* include blood culture, microscopy, and tissue slice, however, their performance is often unsatisfactory. The isolation of *Nocardia* from clinical samples is regarded as the gold standard for diagnosing *Nocardia* disease (21). However, traditional cultivation of *Nocardia* typically requires at least 2 to 7 days, even 4 to 6 weeks (9, 22), which can result in clinical misdiagnosis and missed diagnoses. Many patients with *Nocardia* disease may receive antibiotic treatment prior to sample collection, leading to a significantly low positivity rate (23). Furthermore, the cell wall of *Nocardia* contains a substantial amount of lipid components, particularly mycolic acids, resulting in weak positive acid–fast staining. This often leads to frequent misdiagnosis of *Mycobacterium tuberculosis* infection.

mNGS demonstrates distinct advantages including high-throughput capacity, rapid turnaround time (typically 24–48 h), and high diagnostic accuracy. This technology enables the detection of rare microorganisms (including viruses, bacteria, fungi, and parasites) that are challenging to identify using conventional culture-dependent methods. Notably, mNGS exhibits minimal impact from antibiotic exposure (24–26). Currently, mNGS is recognized as a pivotal diagnostic tool for detecting uncommon pathogens owing to its superior sensitivity and specificity. The clinical implementation of this technology holds significant potential to advance early and precise diagnosis of rare or atypical infectious diseases, thereby mitigating associated mortality rates (27, 28). However, mNGS also has several limitations (29, 30) (1) a single test may cost approximately 550 USD in China, and it is not covered by medical insurance, imposing a significant financial burden on patients; (2) environmental factors, sampling issues, contaminants may lead to false-positive results; (3) lack of standardized workflow validation and quality control, which may impact reproducibility; (4) insensitivity to high-host background and low-biomass samples; (5) inability to distinguish colonization from true infection and (6) no antimicrobial susceptibility data, limiting its utility in guiding optimal antibiotic selection. In summary, although mNGS is a valuable diagnostic technology, its inherent limitations should not be overlooked. Therefore, regardless of whether mNGS results are positive or negative, clinicians should integrate findings from other microbiological tests (culture, serology, PCR), clinical manifestations, and epidemiological history to make sound clinical decisions. Notably, when mNGS is unavailable, clinical laboratories may employ alternative approaches for rapid *Nocardia* identification, including: polymerase chain reaction (PCR), multilocus sequence analysis (MLSA), quantitative real-time PCR (qPCR) and clustered regularly interspaced short palindromic repeats (CRISPR). These approaches also provide critical support for the early identification of *Nocardia* infections. The patient’s, conventional microbiological investigations (blood cultures, CSF cultures, and smears) yielded negative results. N*ocardia otitidiscaviarum* in CSF were rapidly detected by mNGS. Combined with the patient’s occupation and injury history, we believed that *Nocardia otitidiscaviarum* inoculated through this traumatic wound and subsequently disseminated to the CNS. Furthermore, since this patient was immunocompetent and had prolonged soil exposure, the findings may not be applicable to all immunocompetent cases.

Cephalosporins, such as ceftriaxone and cefotaxime, are frequently employed empirically to treat intracranial infections. However, *Nocardia* typically exhibits a high resistance rate to beta-lactam antibiotics, which can lead to the failure of empirical anti-infection treatment and subsequently increase patient mortality (31). Currently, TMP-SMZ is recommended as the first-line treatment for *Nocardia* disease. In contrast, meropenem, imipenem, amikacin, linezolid, and tetracycline antibiotics are regarded as alternative options for *Nocardia* (32). The management of *Nocardia* infections is individualized based on patient-specific factors, utilizing either monotherapy or combination antimicrobial regimens (33). For patients with uncomplicated cutaneous involvement, the standard therapy typically consists of TMP-SMZ or linezolid as monotherapy. In cases of pulmonary involvement without CNS complications, treatment may include TMP-SMZ or linezolid, which can be administered either alone or in combination with other agents. For CNS *nocardiosis*, it is recommended to initiate a combination of at least two antibiotics, such as TMP-SMZ, amikacin, imipenem, meropenem, or linezolid. To reduce mortality and relapse rates, antimicrobial therapy for central nervous system nocardiosis should be administered for at least 9 to 12 months, or even longer (14, 34, 35), however, the optimal treatment length remains uncertain (8). In this case, prior treatment with ceftriaxone or meropenem combined with vancomycin failed to control the infection, however, treatment with TMP-SMZ, meropenem, or amikacin resulted in symptom improvement. The patient was discharged on February 22, 2024, transitioning to an oral TMP-SMZ and linezolid regimen. During the subsequent 11-month period, the patient underwent multiple follow-up visits to the hospital, with stable clinical status and CSF analysis results returning to near-normal levels.

## Conclusion

Purulent meningitis caused by *Nocardia otitidiscaviarum* is rare and is associated with a high mortality rate. The clinical features of such infections lack specificity, and traditional diagnostic methods are often time-consuming and yield a low positive rate in identifying the pathogen, which can result in frequent missed or misdiagnoses. This case report demonstrates that immunocompetent patients with intracranial infections, particularly those exhibiting resistance to conventional antimicrobial therapy, must be regarded as high-risk candidates for *Nocardia*-associated cerebral infections. mNGS plays a critical role in early pathogen identification and guiding subsequent management strategies. Timely and accurate diagnosis combined with prompt targeted antimicrobial therapy has been demonstrated to significantly enhance the cure rate of such patients. TMP-SMZ is the first-line treatment for cerebral *nocardiosis* and is frequently combined with 2 to 3 additional drugs that effectively penetrate the blood–brain barrier to mitigate high mortality rates.

## Data Availability

The raw data supporting the conclusions of this article will be made available by the authors, without undue reservation.
