# Supplementary figures and images for: Nocardia otitidiscaviarum meningitis in an immunocompetent patient diagnosed by metagenomic next-generation sequencing: a case report
Source: Front Med (Lausanne). 2025 Aug 11;12:1588977. doi: 10.3389/fmed.2025.1588977 (PMC12375495; doi:10.3389/fmed.2025.1588977)

A

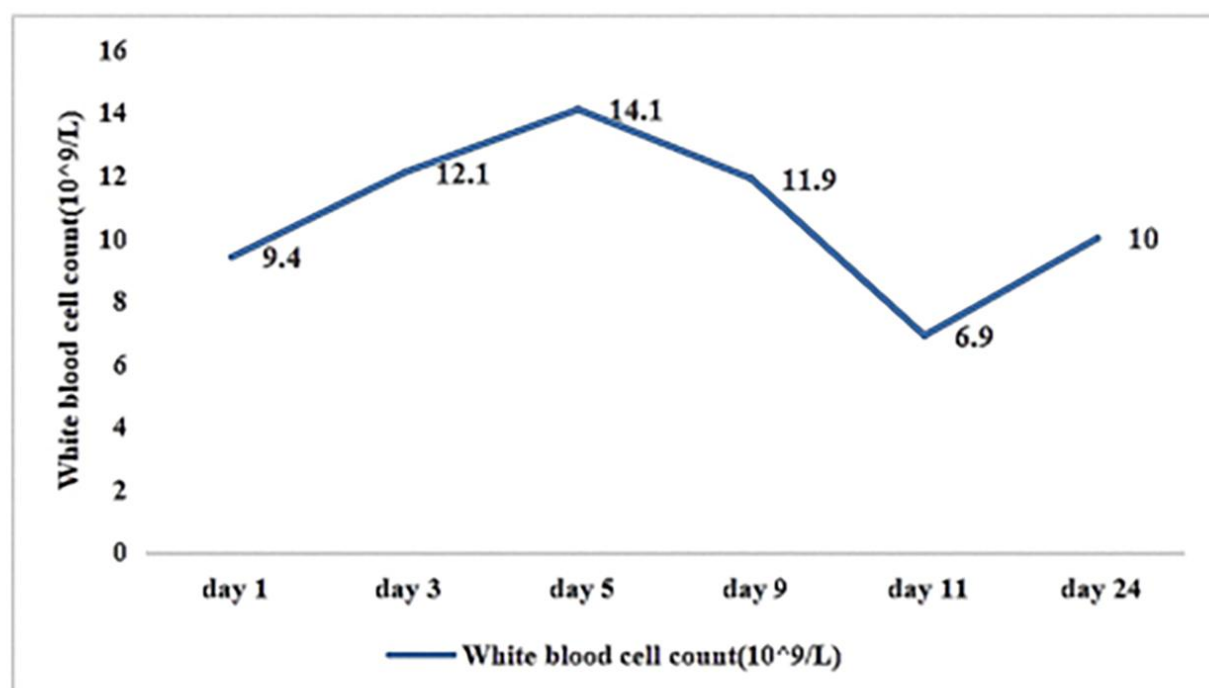

B

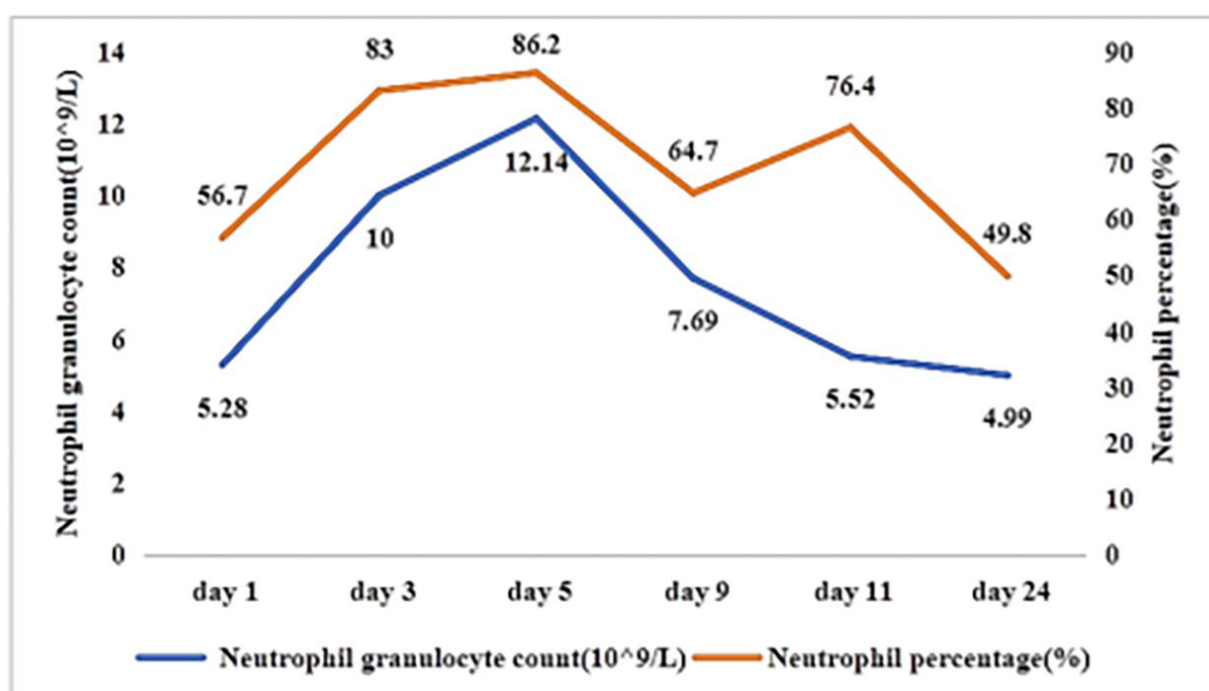

C

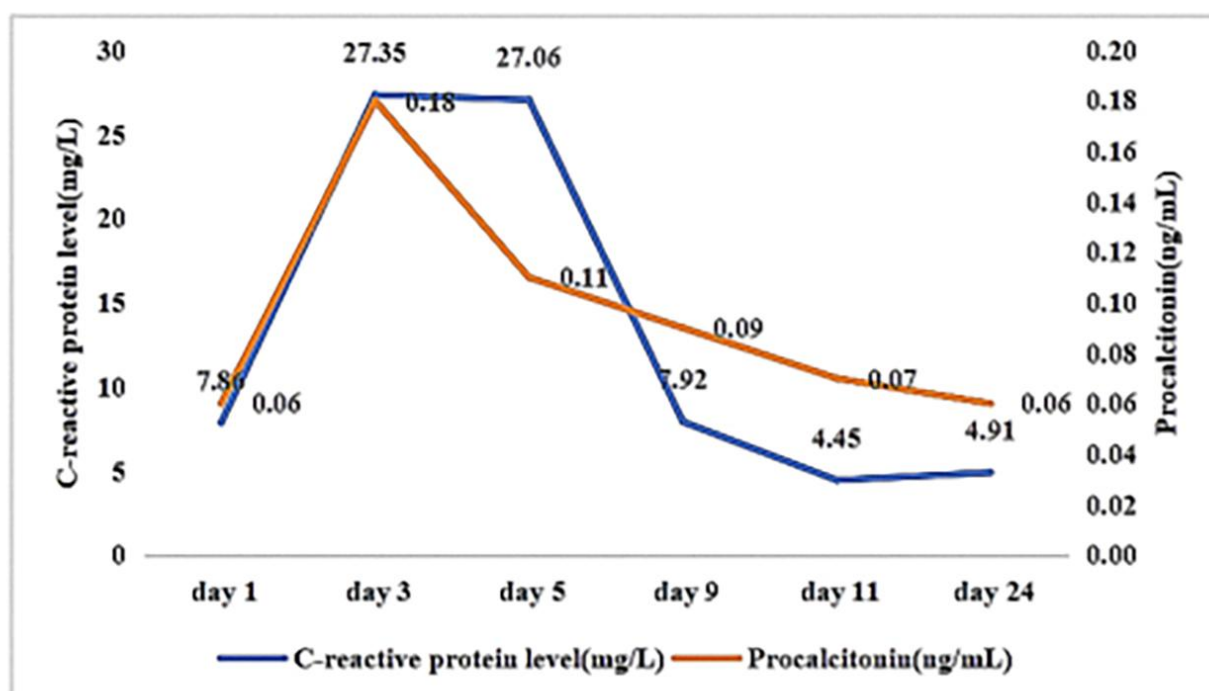

Supplement: Supplementary file 1 [file Data_Sheet_1.pdf]
